# White spot syndrome virus impact on the expression of immune genes and gut microbiome of black tiger shrimp *Penaeus monodon*

**DOI:** 10.1038/s41598-023-27906-8

**Published:** 2023-01-18

**Authors:** Thapanan Jatuyosporn, Pasunee Laohawutthichai, Juan Pablo Ochoa Romo, Luigui Gallardo-Becerra, Filiberto Sánchez Lopez, Anchalee Tassanakajon, Adrian Ochoa-Leyva, Kuakarun Krusong

**Affiliations:** 1 Center of Excellence in Structural and Computational Biology, Department of Biochemistry, Faculty of Science, Chulalongkorn University, Bangkok, 10330 Thailand; 2 Center of Excellence for Molecular Biology and Genomics of Shrimp, Department of Biochemistry, Faculty of Science, Chulalongkorn University, Bangkok, 10330 Thailand; 3 Departamento de Microbiología Molecular, Instituto de Biotecnología (IBT), Universidad Nacional Autónoma de México (UNAM), Av. Universidad #2001, Col. Chamilpa, 62210 Cuernavaca, Morelos Mexico

**Keywords:** Antimicrobial responses, Innate immunity, Marine biology

## Abstract

The gut microbiome plays an essential role in the immune system of invertebrates and vertebrates. Pre and pro-biotics could enhance the shrimp immune system by increasing the phenoloxidase (PO), prophenoloxidase (ProPO), and superoxide dismutase activities. During viral infection, the host immune system alteration could influence the gut microbiome composition and probably lead to other pathogenic infections. Since the JAK/STAT pathway is involved in white spot syndrome virus (WSSV) infection, we investigated the intestine immune genes of STAT-silenced shrimp. During WSSV infection, expression levels of *Pm*Vago1, *Pm*Doral, and *Pm*Spätzle in *Pm*STAT-silenced shrimp were higher than normal. In addition, the transcription levels of antimicrobial peptides, including crustin*Pm*1, crustin*Pm*7, and *Pm*PEN3, were higher in WSSV-challenged *Pm*STAT-silenced shrimp than the WSSV-infected normal shrimp. Meanwhile, *Pm*STAT silencing suppressed *Pm*ProPO1, *Pm*ProPO2, and *Pm*PPAE1 expressions during WSSV infection. The microbiota from four shrimp tested groups (control group, WSSV-infected, *Pm*STAT-silenced, and *Pm*STAT-silenced infected by WSSV) was significantly different, with decreasing richness and diversity due to WSSV infection. The relative abundance of *Bacteroidetes, Actinobacteria*, and *Planctomycetes* was reduced in WSSV-challenged shrimp. However, at the species level, *P. damselae,* a pathogen to human and marine animals, significantly increased in WSSV-challenged shrimp. In constrast, *Shewanella algae*, a shrimp probiotic, was decreased in WSSV groups. In addition, the microbiota structure between control and *Pm*STAT-silenced shrimp was significantly different, suggesting the importance of STAT to maintain the homeostasis interaction with the microbiota.

## Introduction

The gut microbiome plays an essential role in the organism’s growth, development, and immunity^1–3^. In addition, the gut microbiome improves the immune response, nutrient absorption, and homeostasis maintenance^4,5^. It is generally accepted, for different organisms, that a higher diversity of the microbiota is associated with a healthy host condition. However, in shrimps, there are examples of low and high diversities associated with diseases^6,7^, so the direct association of lower diversity with the disease is still under discussion^8,9^. For example, Cornejo-Granados et al., (2017), found increased *Aeromonas taiwanensis*, *Simiduia agarivorans*, and *Photobacterium angustum*, which could be disease-specific bacteria during the early development of acute hepatopancreatic necrosis disease (AHPND) in *Litopenaeus vannamei*. Furthermore, AHPND reduced the Shannon diversity index of *L. vannamei* stomachs, where *Vibrio* and *Candidatus Bacilloplasma* were predominant populations, by 53.6%^6^. In addition, at the genus level, *Photobacterium*, *Propionigenium,* and *Arcobacter* were significantly increased, while *Candidatus*, *Bacilloplasma,* and *Flavobacterium* decreased in WSSV-infected *L. vannamei*^10^.

Moreover, pre- and probiotic diets can improve the gut microbiome and benefit the shrimp’s health. For example, *L. vannamei* fed with a *Lactobacillus plantarum* mixed diet improved the activities of phenoloxidase (PO), prophenoloxidase (ProPO), and superoxide dismutase (SOD), as well as increased the clearance efficiency of *Vibrio alginolyticus* and *V. harveyi*^11,12^*.* Furthermore, *L. vannamei* fed with probiotic *Clostridium butyricum*has improved the expression of the host immune-related genes, including ProPO, lipopolysaccharide and β-1,3-glucan binding protein, lysozyme, crustin, and SOD which might enrich the beneficial bacteria such as *Bacillus*, *Clostridium*, *Lachmoclostridium*, *Lachnospiraceae*, and *Lactobacillus*^13^. This finding supported that the microbiome plays a vital role in the training and developing of the shrimp’s innate immune system. In contrast, the immune system controls the maintenance of host-microbe symbiosis^14^.

Shrimp immunity plays an essential role in microorganism invasion and balances host-microbe symbiosis. Like other invertebrates, pattern-recognition proteins (PRPs) act as an invading censor and activate intracellular signaling, stimulating humoral immune responses^15^. Shrimp humoral responses are the front-line defense against pathogens. Several signaling immune pathways are involved depending on the type of pathogen^16^. The Toll, immune deficiency (IMD), and JAK/STAT pathways regulate the immune response of invertebrates^17^. The Toll pathway responds to Gram-positive bacteria with Lys-type peptidoglycan, fungi, and some viruses such as white spot syndrome virus (WSSV)^18–21^. The microbial inducer-PRP complex activates the proteolytic cascade, resulting in the active Spätzle activating the Toll receptor^20^. The activated Toll receptor leads three cytoplasmic proteins, MyD88, Tube, and Pelle, to form the heterotrimeric complex. The activated Pelle can dissociate the Castus-Dorsal complex by phosphorylation^22^. The free Dorsal named NF-ƘB transcription factor translocates into the nucleus and up-regulates antimicrobial peptide (AMP) genes^23,24^. Meanwhile, the IMD pathway acts against Gram-negative bacteria, some Gram-positive *Bacilli* with the mesodiaminopimelic acid-type (DAP-type) PNGs, and some RNA viruses such as the yellow head virus (YHV)^25–27^. The pathogens were recognized by membrane-bound PRPs and stimulated IMD cascade through TAK1, TAB1, and TAB2 to activate IKK/Relish branches^28–32^. In shrimp, IKKβ and IKKε have been identified^33,34^.

**Table 1:** Alpha diversity metrics for all the treatments. Species richness was measured using Chao2 and observed species index. Species diversity was calculated using Phylogenetic diversity (PD) and the Shannon diversity index.

	Chao1	Observed species	PD	Shannon diversity index
PBS	486.78 ± 25.29^a^	404.41 ± 26.82^a^	25.87 ± 1.57^a^	5.56 ± 0.46^a^
*Pm*STAT dsRNA	491.64 ± 5.23^a^	401.52 ± 16.15^a^	24.94 ± 0.81^a^	5.17 ± 0.26^a,b^
*Pm*STAT dsRNA + WSSV	361.32 ± 13.74^b^	268.41 ± 13.93^b^	18.70 ± 0.75^b^	3.46 ± 1.15^b,c^
WSSV	382.73 ± 12.57^b^	275.65 ± 11.42^b^	20.52 ± 1.14^b^	2.26 ± 0.39^c^

Different superscript lowercase letters in the same column indicate significant differences among conditions (*P* < 0.01).

**Table 2:** Pairwise distance between the centroids for all the treatments.

	PBS	*Pm*STAT dsRNA	WSSV	*Pm*STAT dsRNA + WSSV
PBS	–			
*Pm*STAT dsRNA	0.510	–		
WSSV	0.659	0.537	–	
*Pm*STAT dsRNA + WSSV	0.628	0.495	0.334	–

The JAK/STAT pathway is activated when the secreted cytokine molecule interacts with JAK/STAT receptor. Then, JAK is activated and introduced to STAT activation by phosphorylation. The active STAT translocates into the nucleus and promotes the transcription of antiviral and immune genes^35–37^. In shrimp, it has been proven that the JAK/STAT pathway could be controlled by the IRF-Vago-JAK/STAT pathway manner, which is similar to the IRF-IFN-JAK/STAT pathway axis of vertebrates^38^. However, WSSV could hijack the JAK/STAT pathway by activating STAT to promote transcription of WSSV immediate-early gene 1 (IE1) and late gene envelope protein 28 (VP28) during WSSV infection^16,39^. The *Lv*DOME, JAK/STAT receptor, or *Lv*STAT silenced shrimp showed reduced WSSV copy numbers and mortality during WSSV infection^16,39–42^. So far, there is no study on the gut microbiome of *Pm*STAT-silenced shrimp.

In vertebrates, the gut microbiome composition and host gene expression are associated. For example, Germ-free mice have lower Cytochrome P450 3a subfamily and transporter genes than normal mice, causing a decrease in the detoxification capability of the germ-free host^43^. In zebrafish, the expression of the hepatocyte nuclear factor 4A was suppressed by the microbiome, leading to the inhibition of host inflammatory pathways^44^. This suggests that lacking host transcription factors impacts the expression of downstream genes and microbiome structure. However, the relationship between immunity and gut microbiome in shrimp is unclear.

In this study, we investigate the shrimp gut microbiome, shrimp immunity, and immune-related genes during WSSV infection in *Pm*STAT-silenced shrimp to fulfill the understanding of host–pathogen-microbiome interactions. Understanding of host-WSSV-microbiome interactions might provide an improved WSSV prevention strategy and the knowledge of bacterial changes associated with a viral infection in a *Pm*STAT-silenced host.

## Results

### *Pm*STAT dsRNA successfully suppressed *Pm*STAT transcript and lower WSSV IE1

**Figure 1:**
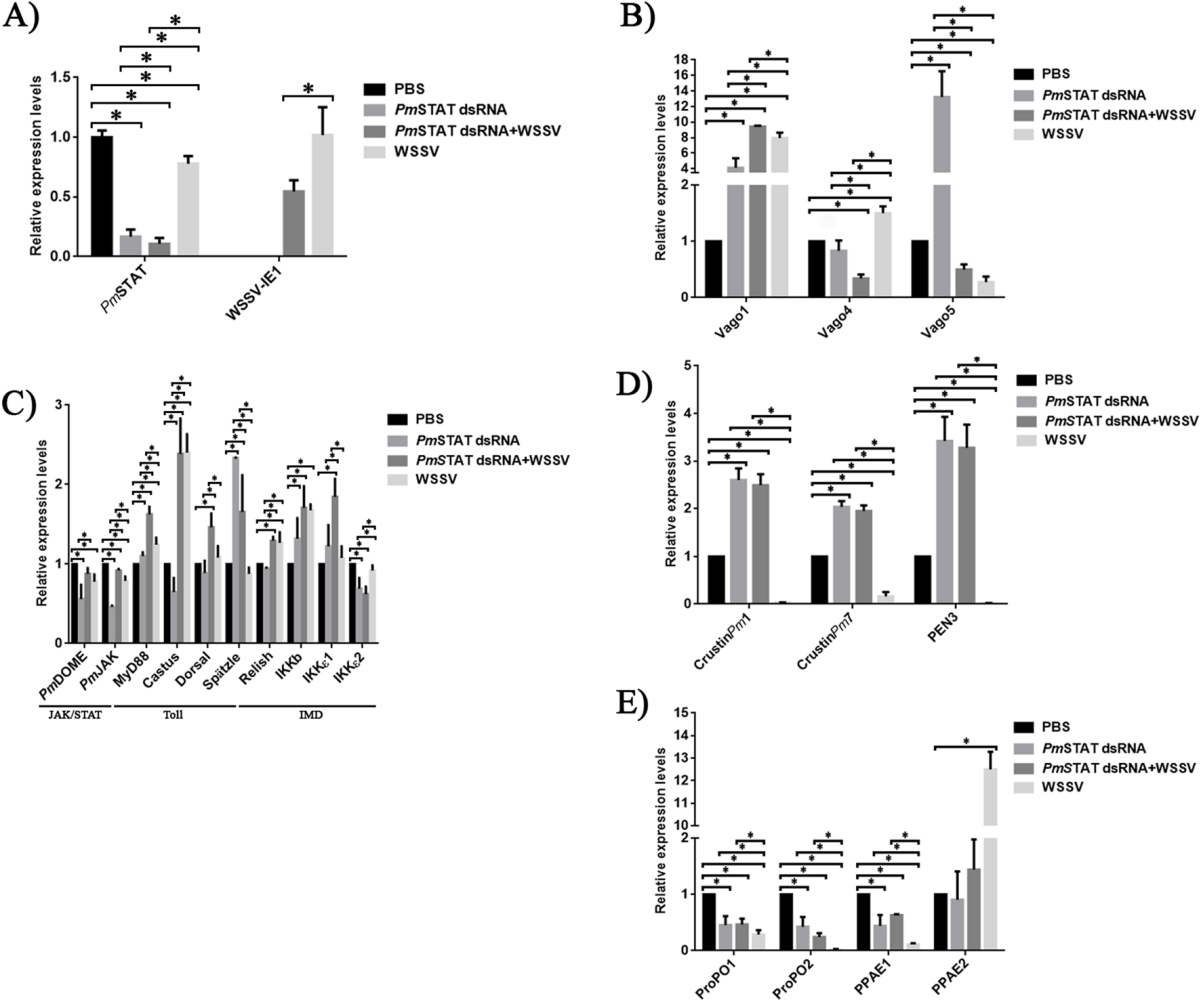
(**A**) *Pm*STAT and IE1 transcription levels in shrimp intestine at 24 h post-WSSV injection. *Pm*STAT was suppressed by injecting *Pm*STAT dsRNA 10 µg per shrimp’s gram, then 16 h later, followed by the same amount with the first injection. The control group was injected with PBS instead *Pm*STAT dsRNA. WSSV was injected at 24 h after the 2nd *Pm*STAT dsRNA injection. Shrimp intestines were collected at 24 h post-infection. In addition, we investigated the transcription levels of immune-related genes upon *Pm*STAT silencing during WSSV infection. (**B**) Transcription levels of interferon-like gene (*Pm*Vago 1, 4, and 5). (**C**) Expression levels of immune-related genes, including JAK/STAT pathway (*Pm*DOME and *Pm*JAK), Toll pathway (MyD88, Castus, Dorsal, and Spätzle), IMD pathway (Relish) and inhibitor of kappa B kinase (IKKβ, IKKε1, and IKKε2). Moreover, Transcription levels of immune genes were also observed. (**D**) Transcription levels of antimicrobial peptide and (**E**) phenol oxidase cascade.

*P. monodon* intestines were collected from PBS-injected, *Pm*STAT-silenced, WSSV-challenged, and *Pm*STAT-silenced + WSSV challenged groups. Each group contained three replicates (n = 3), and each replicate was a pool of three shrimp intestines. From those, the DNA and total RNA were extracted using typical protocols. After that, quantitative real-time RT-PCR was performed to examine *Pm*STAT silencing efficiency and WSSV IE1 transcription levels. The *Pm*STAT expression levels in the shrimp intestines were successfully suppressed in *Pm*STAT dsRNA injection groups. Meanwhile, transcription levels of the IE1 gene in the intestines of *Pm*STAT knockdown shrimps were significantly lower than normal shrimps challenged by WSSV (Fig. 1A). This implied that *Pm*STAT knockdown reduced WSSV infection.

## Effect of *Pm*STAT silencing on immune relate genes

The total RNA of *P. monodon* intestines was extracted and subjected to qRT-PCR to investigate the expression of immune-related genes, including JAK/STAT, Toll, IMD, cytokine, phenol oxidase, and antimicrobial peptide. The transcription levels of Vago, an IFN-like antiviral cytokine, responded to *Pm*STAT silencing and WSSV challenge (Fig. 1B). The Vago1 was strongly up-regulated in *Pm*STAT dsRNA, *Pm*STAT dsRNA + WSSV and WSSV challenged group. It is worth noting that the expression level of Vago1 in *Pm*STAT dsRNA + WSSV shrimp was significantly higher than that in *Pm*STAT dsRNA and WSSV-challenged group. In contrast, Vago4 was up-regulated in normal shrimp infected by WSSV but down-regulated in *Pm*STAT-silenced shrimp infected by WSSV. As shown in Fig. 1B, *Pm*STAT silencing significantly enhanced Vago5 transcription level, but the WSSV challenge reduced Vago5 transcripts in both normal and *Pm*STAT-depleted shrimp.

In *Pm*STAT-silenced shrimp, the expression levels of the JAK/STAT genes, including *Pm*DOME and *Pm*JAK, were decreased. In constrast, their expression levels were increased upon WSSV infection (Fig. 1C). Regarding the Toll pathway, silencing of *Pm*STAT enhanced the *Pm*Spätzle expression level more than two-fold, compared to normal shrimp but showed fewer effects on the transcription levels of MyD88, Castus, and Dorsal. The expression level of *Pm*Spätzle in *Pm*STAT-silenced shrimp was dropped upon WSSV infection (Fig. 1C). Meanwhile, the transcription levels of Relish, a transcription factor in the IMD pathway, in normal and *Pm*STAT-silenced shrimp slightly increased in response to WSSV infection (Fig. 1C). In this work, the expression level of the inhibitor of kappa B kinase, which plays an essential role in the IKK-NF-ƘB signaling cascade, was also investigated. The expression level of *Pm*IKKβ was increased in response to *Pm*STAT depletion and WSSV infection (Fig. 1C). *Pm*IKKε1 transcript was increased in both non-infected and WSSV-infected *Pm*STAT-silenced shrimp, compared to the normal shrimp, while *Pm*IKKε2 transcript was decreased.

Antimicrobial peptides, including crustin*Pm*1, crustin*Pm*7, and peneaidin3, responded to *Pm*STAT dsRNA. At the same time, the WSSV infection strongly suppresed them (Fig. 1D). Meanwhile, transcription levels of phenol oxidase cascade, including ProPO1, ProPO2, and phenol oxidase activating enzyme 1 (PPAE1), were significantly suppressed by either *Pm*STAT dsRNA or WSSV. However, PPAE2 expression was promoted by the WSSV challenge (Fig. 1E).

## Shrimp intestinal microbiome

A total of 1,544,254 sequences were obtained from 12 libraries, consisting of 4 conditions in triplicate (PBS, *Pm*STAT dsRNA, *Pm*STAT dsRNA + WSSV, and WSSV). The average sequencing read was 128,687 per sample, ranging from 101,376 to 162,622. The operational taxonomic units (OTUs) were assigned to a 97% sequence similarity. The reads assigned to OTUs (without singletons and low abundance OTUs (0.005%)) ranged from 60,557 (46.66%) to 94,696 (75.07%), which averaged read were 66,909.33 (49.12%), 82,021.00 (57.14%), 83,628.67 (69.93%) and 74,944.00 (60.82%) in PBS, *Pm*STAT dsRNA, WSSV, and *Pm*STAT dsRNA + WSSV, respectively (Supplementary Information, Tables S1and S2). The percentage of the classified taxonomic level is shown in Table S3. The classified percentage ranged from 45.26% to 91.84% at the genus level. Each condition shared 151 genera and 69 species in common. Meanwhile, the unique bacteria found in species levels in each condition, including PBS, *Pm*STAT dsRNA, *Pm*STAT dsRNA + WSSV, and WSSV, were 49, 41, 13, and 20, respectively (Supplementary Information, Fig. S1).

**Figure 2:**
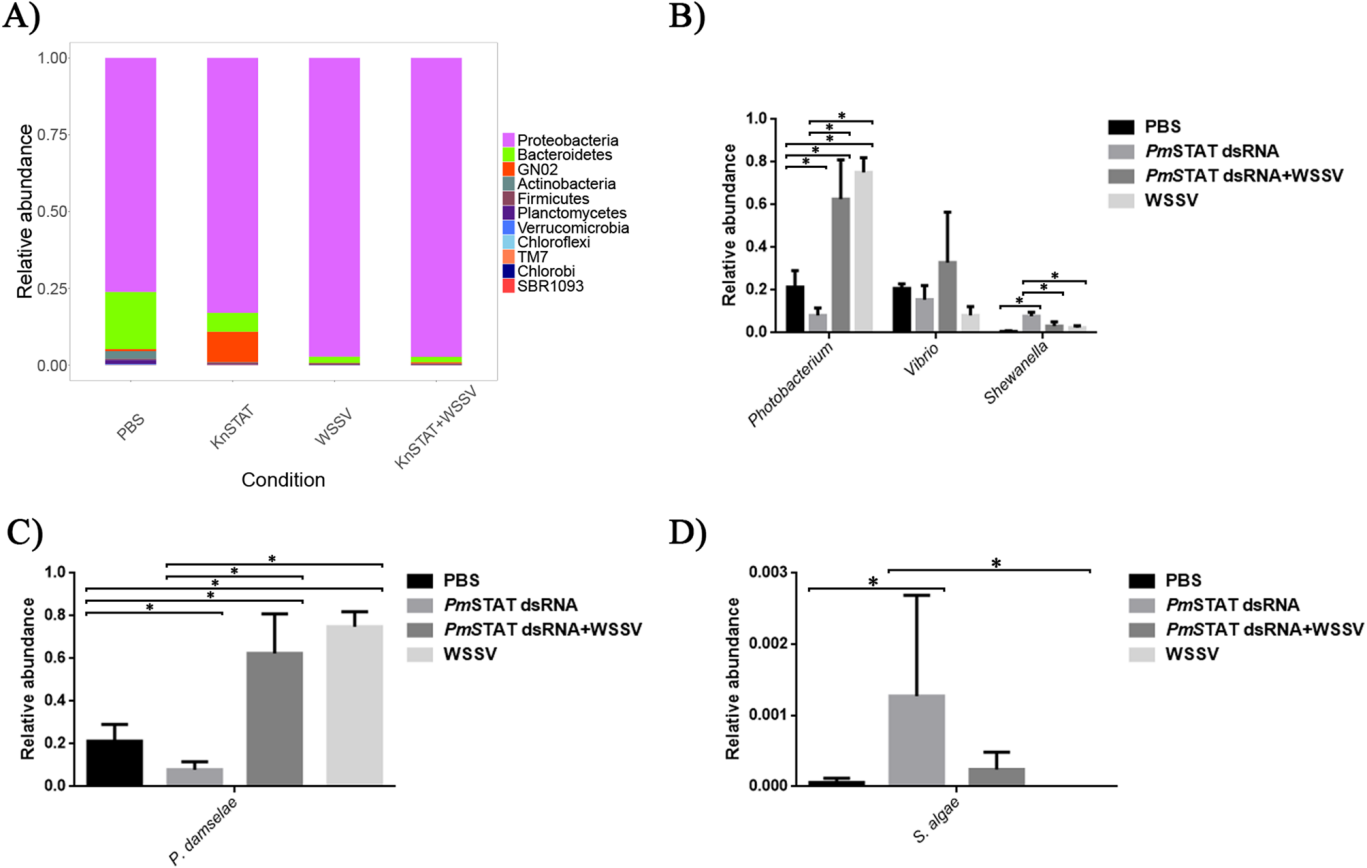
(**A**) Intestinal bacterial composition in PBS, *Pm*STAT-silenced, WSSV-challenged, and *Pm*STAT-silenced combined WSSV challenged on phylum level. The most abundant genera (**B**) and species level (**C**). The relative abundance of potential probiotic, *Shewanella algae* (**D**).

The dominant phyla in all samples were *Proteobacteria*, followed by *Bacteroidetes*, *Actinobacteria*, and *Planctomycetes* (Fig. 2A). However, the relative abundance of phyla *Bacteroidetes*, *Actinobacteria*, and *Planctomycetes* were reduced in WSSV and *Pm*STAT silenced intestine. Interestingly, *Proteobacteria* was significantly increased in WSSV-challenged shrimp (*P* < 0.01). The rest of the phyla, including *Bacteroidetes*, *Actinobacteria*, *Planctomycetes*, *Firmicutes*, *Verrucomicrobia, GN02* (*Gracilibacteria*), *Chloroflexi*, and *TM (Saccharibacteria),* were significantly reduced in WSSV challenged and also in the *Pm*STAT dsRNA + WSSV intestine. The highest relative abundance was *Photobacterium* at the genus level, followed by *Vibrio* and *Shewanella* (Fig. 2B). The *Photobacterium* was increased in both WSSV-challenged shrimps. Meanwhile, the relative abundance of *Vibrio* was similar in all conditions, and *P. damselae* was significantly increased in both WSSV-challenged shrimps (Fig. 2C). The Linear discriminant analysis effect size (LEfSe) analysis showed that the most LDA scores in each condition, including PBS, *Pm*STAT dsRNA, WSSV and *Pm*STAT dsRNA + WSSV was genera *Cohesibacter, Shewanella, Roseivirga* and *Marivita*, respectively (Fig. S2). Moreover, potential shrimp probiotics were identified in our samples following the previously reported method^45^. Overall, the relative abundance considering all potential probiotics was not significantly different between the four conditions (Supplementary Information, Fig. S4). The potential probiotics with abundance in our samples at the genus level were *Bdellovibrio*, *Phaeobacter*, *Pseudoalteromonas*, *Rhodobacter*, *Shewanella*, *Streptomyces*, and *Vibrio*. While at the species level, we found *Phaeobacter sp. DCSW07*, *﻿Shewanella algae,* and *Vibrio hepatarius* (Supplementary Information, Fig. S5). Interestingly, when we analyzed the abundance of each of the potential probiotic species separately, we found that *Shewanella algae* significantly increased in *Pm*STAT dsRNA compared to all other three conditions. On the contrary, *S. algae* were depleted in the WSSV group (Fig. 2D); however, the differences were insignificant.

The alpha diversity metrics were calculated to investigate the difference between richness (Chao1 and the observed species index) and diversity (Phylogenetic diversity (PD) and Shannon diversity index) among groups (Table 1, Rarefaction curves are shown in Fig. S6). The unchallenged groups (PBS and *Pm*STAT silenced shrimp) showed higher richness and diversity. Contrarily, the challenged groups (*Pm*STAT silencing with WSSV and WSSV infected) showed lower richness and diversity. Overall, species richness and diversity were reduced in WSSV challenged shrimps and different from the PBS and *Pm*STAT silencing shrimps which were similar.

**Figure 3:**
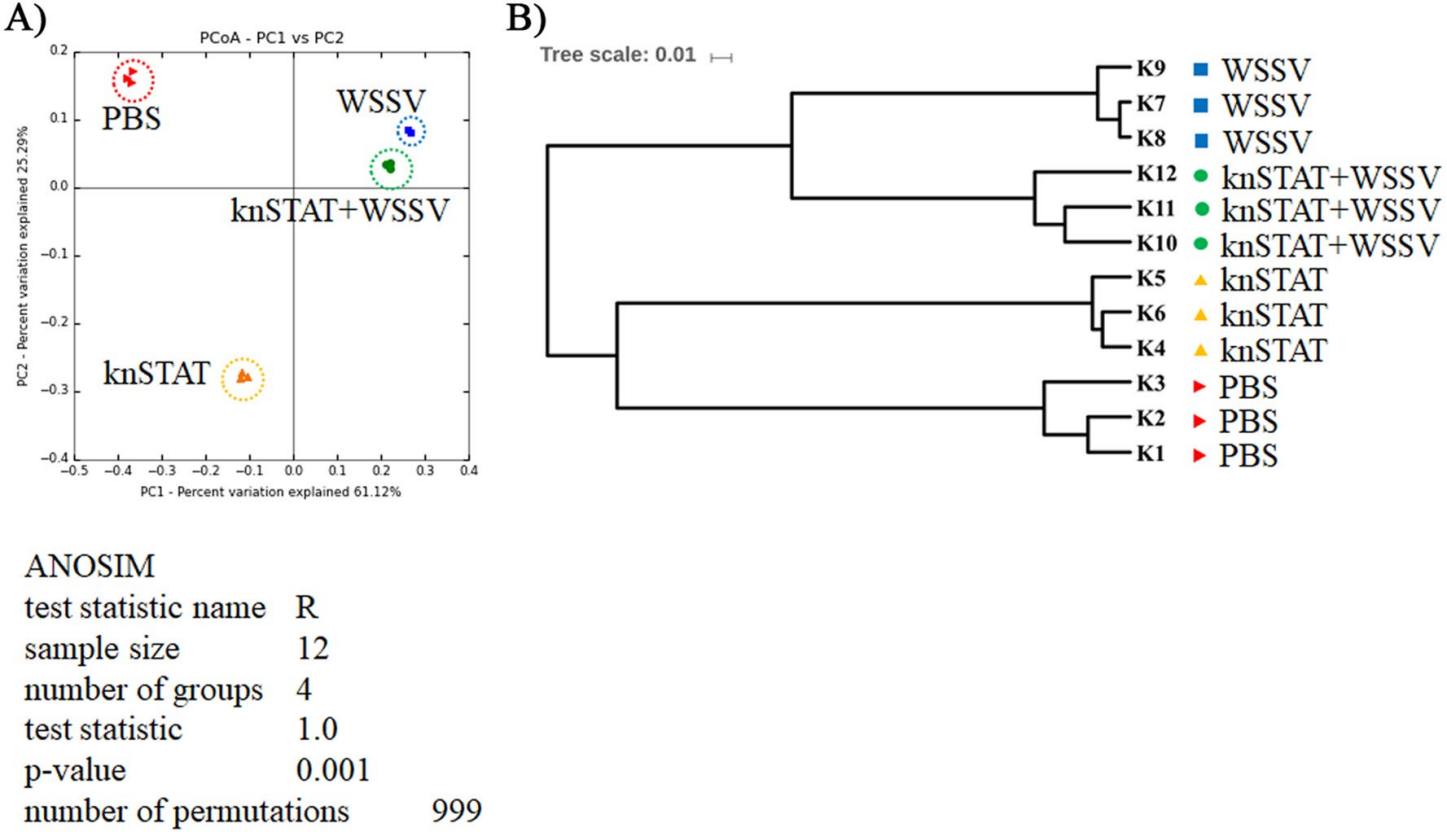
(**A**) Principal coordinate analysis (PCoA) based on unweighted UniFrac distance and (**B**) unweighted UPGMA clustering. The knSTAT refers to *Pm*STAT silencing shrimp.

The differences in intestinal microbial communities among groups were analyzed by beta diversity. Principal coordinate analysis (PCoA) based on unweighted UniFrac distances exhibited that all samples formed four significantly separated clusters (ANOSIM p = 0.001) (Fig. 3A). The distances in the PCoA are shown in Fig. 3A. Thus, we quantitatively determined the centroids for each group of samples and then calculated the distance between the centroids for all the treatments (Table 2). With this analysis, we found several behaviors: First, the most extended separations of the distances between the control (PBS) and all the treatments suggested that the microbiota of all conditions differed significantly from that of the control. Second, the distance of *Pm*STAT dsRNA was more significant Vs. WSSV (0.537) and Vs. *Pm*STAT dsRNA Vs *Pm*STAT dsRNA + WSSV (0.495), suggesting that the *Pm*STAT dsRNA microbiota significantly differed from the WSSV and *Pm*STAT dsRNA + WSSV. Third, the smaller distance between all comparisons was between WSSV Vs. *Pm*STAT dsRNA + WSSV (0.334), suggesting that the microbiota between those two conditions was more similar than the other group comparisons. This also suggested that the most substantial effect on the microbiota composition caused by WSSV infection, rather than *Pm*STAT dsRNA depletion.

Similar clusters were also observed in the UPGMA tree of unweighted UniFrac distances, in which all WSSV-infected shrimps formed one hand of the three, in which the WSSV-infected shrimps clustered separately from the *Pm*STAT with WSSV (Fig. 3B). Contrarily, the non-infected shrimps created another hand (Fig. 3B), in which the PBS shrimps were separated from the *Pm*STAT-silenced shrimps. These findings agreed with the positions observed between the four groups in the unweighted PCoA (Fig. 3A).

## Discussion

Gut microorganisms are essential in host functions, including development, nutrition, immunity, and disease resistance. However, host-pathogen interaction is still unclear. Therefore, we investigated the intestinal bacterial and immune-related transcription profile. Shrimp intestines were collected from WSSV unchallenged and challenged. In addition, we also investigated those profiles in suppressed JAK/STAT, an antiviral pathway in shrimps.

Changes in bacteria composition during WSSV infection may influence the expression of shrimp immunity. In mosquitoes, Vago function as an IFN-like antiviral cytokine. The transcription levels of *Lv*Vago4 and *Lv*Vago5 were up-regulated in WSSV-challenged hemocyte species^38^. Unlike the hemocyte, the transcription level of *Pm*Vago4 was slightly up-regulated, and *Pm*Vago5 was dramatically down-regulated in WSSV challenged intestine. However, *Pm*Vago5 was induced in *Pm*STAT dsRNA shrimp (Fig. 1B). Meanwhile, *Pm*Vago1 was strongly up-regulated by either *Pm*STAT dsRNA or WSSV challenged. It has been reported that *Lv*TCF, a main downstream effector of Wnt signaling, regulates *Lv*Vago1. During WSSV infection in *L. vannamei* hemocytes*,* the transcription levels of *Lv*TCF were increased. However, WSSV produced WSV083 to promote the degradation of *Lv*TCF via the ubiquitin-proteasome pathway, which suppressed transcription levels of *Lv*Vago1^46^. Furthermore, *Lv*IRF, an interferon regulatory factor, regulates the transcription levels of *Lv*Vago4 and *Lv*Vago5. In *L. vannamei* hemocytes, the expression of *Lv*Vago4 and *Lv*Vago5 were inhibited in *Lv*IRF-silenced shrimp. Moreover, cumulative mortality and WSSV copy numbers were increased in *Lv*Vago4- or *Lv*Vago5-silenced shrimp^38^. Unlike *L. vannamei* hemocytes, *Pm*STAT silencing promoted transcription levels of *Pm*Vago1 and *Pm*Vago5 in the *P. monodon* intestine (Fig. 1B). Meanwhile, WSSV infection strongly induced the expression of *Pm*Vago1 and slightly induced the expression of *Pm*Vago5 (Fig. 1B). Moreover, suppression of *Pm*STAT reduced WSSV copy numbers and promoted the transcription levels of *Pm*Vago1 (Fig. 1A and B). This suggests that *Pm*Vago1 might be the immunity frontline against WSSV in the intestine, unlike hemocytes.

Figure 1C showed that *Pm*STAT silencing decreased the transcription levels of *Pm*DOME and *Pm*JAK. When *Pm*STAT-silenced shrimp were challenged by WSSV, the expression of *Pm*DOME and *Pm*JAK was increased, suggesting that WSSV infection altered the JAK/STAT pathway. Since the Dome functionally reduced the WSSV copy number (e.g., replication), presumably, it should be increased when STAT is inhibited. However, the effect of down-regulation of *Pm*Dome in the *Pm*STAT-silenced intestine could suggest novel functions that need more studies.

The expression of *Pm*Dorsal, a transcription factor in the Toll pathway, and *Pm*Spätzle were promoted in the *Pm*STAT dsRNA group (Fig. 1C). Two WSSV microRNA named WSSV-miRNA-N13 and WSSV-miRNA-N23 were identified^47^. They suppressed the expression of *Mj*Dorsal, resulting in the inhibition of *Mj*ALF expression. In this study, *Pm*STAT dsRNA promoted expression of *Pm*Dorsal in WSSV-challenged shrimp (Fig. 1C). Previous study has demonstrated that crustin*Pm*1 is controlled through the Toll signaling pathway while crustin*Pm*7 is mediated via both Toll and Imd pathways^48^. Meanwhile, *Lv*penaeidin3a is regulated through the Toll pathway^49^. The transcription levels of crustin*Pm*1, crustin*Pm*7, and *Pm*penaeidin3 were increased in *Pm*STAT silenced shrimp, while in WSSV-challenged, they were dramatically decreased (Fig. 1D). The ProPO system is one of the important shrimp immunities^50^. *P. monodon* hemocyte is the primary cell that express the proteins of this system, while the intestine could not sense transcription in PCR^51,52^. The *Pm*ProPO1, *Pm*ProPO2, and *Pm*PPAE1 were expressed in low levels (Fig. 1E) and dramatically suppressed in WSSV-challenged shrimp. Interestingly, *Pm*PPAE2 was strongly promoted in WSSV-challenged shrimp. The ProPO and PPAE are important to PO activity. Single or double ProPO silencing decreased PO activity. However, there were no significant differences in PO activity between single or double ProPO silencing^52^. Similar results were shown in single or double PPAE silencing. Moreover, single PPAE silencing promoted the expression of other PPAE^53^. The strongly enhancing *Pm*PPAE2 might result from dramatically suppressed *Pm*PPAE1 by WSSV. However, the expression of *Pm*PPAE2 was not enhanced in *Pm*STAT silencing during WSSV infection. Thus, the *Pm*STAT may be necessary to transcript *Pm*PPAE2.

The antimicrobial peptides, including crustin*Pm*1, crustim*Pm*7, and PEN3, were strongly promoted in *Pm*STAT-silenced and *Pm*STAT-silenced + WSSV groups and significantly suppressed in WSSV-infected group (Fig. 1D). A previous study suggested that *Lv*PEN3 could inhibit WSSV virion internalization into hemocytes^54^. Furthermore, *Fm*PEN3 could hinder the growth of *Micrococcus lysodeikticus*^55^. The stimulation of the host immune system by RNAi could be beneficial to prevent animals from pathogen invasion.

Suppression of *Pm*STAT affected transcription levels of many genes, regardless of WSSV infection. *Pm*Vago1 and *Pm*Vago5 were significantly up-regulated in *Pm*STAT-silenced shrimp (Fig. 1B). *Pm*STAT silencing also promoted the expression of Spätzle (Fig. 1C), crustin*Pm*1, crustin*Pm*7, and PEN3 (Fig. 1D). *Pm*Vago1, crustin*Pm*1, crustin*Pm*7, and PEN3 remained significantly up-regulated in *Pm*STAT-silenced shrimp upon WSSV infection, suggesting that these genes may play a key role against WSSV in *Pm*STAT-silenced shrimp. Furthermore, our results showed that *Pm*Vago1 and PPAE2 were up-regulated up to 8- and 12-fold during WSSV infection, respectively (Fig. 1B and 1E). Thus, *Pm*Vago1 and PPAE2 could be considered biomarkers for WSSV infection.

The dominant bacteria phyla were *Proteobacteria* in all shrimp conditions (Fig. 2A). This is similar to previous studies in many crustaceans, including *Callinectes sapidus*, *Eriocheir sinensis*, *Macrobrachium nipponense*, *Penaeus monodon*, *Litopenaeus vannamei* and copepod species^56–60^. However, the Proteobacteria population was significantly increased in WSSV-challenged shrimps, similar to the shift of intestinal microbiota reported in *E. sinensis* and *L. vannamei*^10,56^. Additionally, these phyla were the most abundant and increased in shrimps with AHPND^60^. At the genus level, the abundance of *Photobacterium* was significantly increased in WSSV-challenged *P. monodon*, which is similar to the observed in WSSV and AHPND-challenged *L. vannamei*^10,60^ (Fig. 2B). At the species level, *P. damselae* was the most abundant in WSSV-infected shrimp, and *P. damselae* subsp. *damselae* has been associated with massive mortality of cultured *L. vannamei*^61^. Moreover, *P. damselae* subsp. *damselae* is also associated with infections in marine animals, including turbot (*Psetta maxima*), rainbow trout (*Oncorhynchus mykiss*), ovate pompano (*Trachinotus ovatus*), eel (*Anguilla reinhardtii*), sea bream (*Sparus aurata*), and mud crab (*Scylla* *paramamosain*)^62–66^. The *P. damselae* subsp. *damselae* infecting *S. paramamosain* and *L. vannamei* showed hepatopancreatic tubules were necrotic^61,66^. The mortality of *P. damselae* subsp. *damselae* infecting *L. vannamei* depended on bacterial infection dosage^61^. Therefore, it is possible that *P. damselae* subsp. *damselae* might be an opportunistic pathogen in shrimp during the WSSV invasion. Moreover, the probiotic bacteria, *S. algae*, was increased in *Pm*STAT dsRNA compared to PBS and two WSSV groups (Fig. 2D). In *L. vannamei* juveniles, *S. algae* was fed as a probiotic for 60 days, improving shrimp body weight and transcription levels of β-1,3-glucan-binding protein (LGBP) which boosted survival rate against *V. parahaemolyticus*^67^. In addition, the *Lv*LGBP dsRNA-treated *L. vannamei* caused shrimp to be more susceptible to *V. parahaemolyticus* or WSSV^68^. Furthermore, r*Pm*LGBP improved the in vitro phenoloxidase (PO) activity of hemocyte suspensions^18^.

The interaction between bacteria inside the community is complex. For example, the genome sequencing of *S. algae*, isolated in France, revealed bacteriocins and antimicrobial peptides^69^. The *S. algae* was increased, while *P. damselae* subsp. *damselae* was decreased in *Pm*STAT-silenced shrimp (Fig. 2C and 2D). The antimicrobial peptides in the Toll pathway, including crustin*Pm*1, crustim*Pm*7, and PEN3, were strongly promoted in *Pm*STAT-silenced groups and significantly suppressed in the WSSV infected group (Fig. 1D). Thus, these antimicrobial peptides could reduce the population of *P. damselae* subsp. *damselae* in *Pm*STAT-deprived shrimp, giving an advantage in defense against WSSV. These antimicrobial peptides, however, did not kill or inhibit the probiotic *S. algae* (Fig. 2D)*.*

The WSSV-challenged shrimp (*Pm*STAT dsRNA + WSSV and WSSV) revealed low richness and diversity (Table 1). Likewise,  the bacterial diversity was reduced in shrimp with white feces syndrome (WFS) challenged with *V. harveyi*^7,70^. Moreover, the intestines of healthy shrimps also have lower diversity than diseased shrimps with AHPND^60^. Notably, no changes in microbial diversity in shrimps infected with WSSV and cotton shrimp-like disease were reported^10,71^. Thus the association of greater diversity with better host health in shrimps is, until now, under discussion^8^, and more studies are necessary to understand the role of the microbial ecosystem in health and disease in aquatic organisms. The UPGMA clustering and PCoA analysis using the unweighted UniFrac distances revealed clusters separating the PBS and WSSV-challenged shrimps (Fig. 3). A similar separation of the microbiota from the control by WSSV infection was also observed in *L. vannamei*^10^. Interestingly the microbiota changes were minimal when we compared the *Pm*STAT silencing and STAT silencing + WSSV (Fig. 2 and Table 2). This suggests that changes in the microbiota by the *Pm*STAT silencing were independent of the WSSV infection.

In a murine model, respiratory viral infections induce secondary bacterial infections. The antiviral immune responses induced by influenza are associated with changes in the microbiota in the respiratory and gastrointestinal tract^72^. The inflammation of the tissues promotes the secretion of type I and II interferons (IFNs), increasing Proteobacteria and Bacteroidetes in the gut^73,74^. Meanwhile, the depletion of type I IFN-alpha/beta receptor in mice improved clearance of secondary *Streptococcus pneumoniae* infection during influenza infection^75^. However, virus infection also promotes probiotic bacteria. For example, *Lactobacillales* were enriched in HIV patients^76^. Moreover, bacteria diversity in HIV patients was higher than in the seronegative group^77^, suggesting that the host immune response shifts the host microbiome. Understanding the interaction between host–pathogen-microbiome could improve strategies to prevent diseases.

Taken together, the shift of immune and immune-related genes could change the bacteria composition in the shrimp intestine, demonstrating the importance of host-microbiota interactions in understanding the diseases. Interestingly, only silencing the *Pm*STAT caused drastic effects on the microbiota structure compared to the PBS (Fig. 3A), suggesting the importance of the host gene expression to maintain the homeostasis interaction with the microbiota. However, there are limited examples of this relationship in aquatic animals. For instance, in kuruma shrimp (*Marsupenaeus japonicus*) the silencing of CTL33 expression led directly to intestinal dysbiosis, tissue damage, and shrimp death^78^, suggesting a fine regulation through the evolution of the intestinal microbiota homeostasis in invertebrates. In this regard, it has also been observed that scallop antimicrobial peptides and proteins are implicated in maintaining microbial homeostasis and are critical molecules in orchestrating host-microbiota interactions^79^. Our work opens the possibility of using gene silencing to understand the relationship between shrimp microbiota and the host in the absence and diseases.

### Methods

This study was conducted under the ethical principles and guidelines according to the animal use protocol 1923021 approved by Chulalongkorn University Animal Care and Use Committee (CU-ACUC). The biosafety guidelines were reviewed and approved by the Institutional Biosafety Committee of Chulalongkorn University (SC-CU-IBC-004–2018). This study was carried out in compliance with the ARRIVE guidelines.

## In vitro double-stranded RNA *Pm*STAT synthesis

Primer pairs used in this experiment are shown in Supplementary Information, Table S4^39^. *P. monodon* hemocyte cDNA was amplified using *Pm*STAT DNA fragment containing T7 promotor in the following conditions: 94 °C for 3 min (denaturation), followed by 35 cycles of 94 °C for 30 s, 60 °C for 30 s and 72 °C for 30 s, and a final extension at 72 °C for 10 min. The PCR products were analyzed by 2% agarose gel electrophoresis and were purified from the agarose gel using GeneHlow™ Gel/PCR kit (Geneaid). The purified PCR products were then used as a template for in vitro transcription using T7 RiboMAX™ Express Large-Scale RNA Production System (Promega) according to the manufacturer’s protocol.

## *Pm*STAT silencing shrimp

The black tiger shrimp, *P. monodon*, of average 3.12 ± 0.17 g bodyweight, were obtained from Charoen Pokphand Farm in Chanthaburi Province, Thailand. Shrimp were acclimated in 120L tanks at ambient temperature and maintained in aerated water with a salinity of **20** ppt for at least one week before beginning the experiments. *P. monodon* was injected twice with *Pm*STAR dsRNA (10 µg per g shrimp) at 0 and 16 h after the first injection. Shrimp’s intestines were collected at 24 h post-second injection. Total RNA was extracted using Tissue Total RNA mini kit (Favorgen, Taiwan). The first-strand cDNA was synthesized using the first-strand cDNA Synthesis Kit (Thermo Fisher, USA) according to manufacturer’s protocol. The knockdown efficiency was measured by qRT-PCR using EF-1α as internal control and calculated by the 2^−∆∆CT^ method^80^.

## Sampling

Shrimp were divided into four groups, PBS-injected, *Pm*STAT-silenced, WSSV-challenged, and *Pm*STAT-silenced combined with WSSV-challenged. Shrimp were cultured in 20L tanks (1 tank per group) at ambient temperature and maintained in aerated water with a salinity of 20 ppt. Each group contained nine shrimp. In the *Pm*STAT silenced groups, shrimp were injected with *Pm*STAT dsRNA 10 µg per gram shrimp body weight, and then after 16 h, they were injected with the same amount of dsRNA. Meanwhile, PBS was injected into the shrimp control set. Approximately 6 × 10^6^ viral copies of WSSV (quantification described in Fernando et al*.*^81^) were injected 24 h after the 2nd *Pm*STAT dsRNA injection. Shrimp’s intestines were collected at 24 h post-WSSV challenge and immediately processed DNA and RNA extraction. Each group contained nine shrimps, and each replicate was pooled from 3 shrimp intestines.

## Total RNA and quantitative real-time PCR

Total RNA was isolated by the Tissue Total RNA mini kit (Favorgen, Taiwan) according to the manufacturer’s protocol. An equal amount of total RNA (500 ng) from each sample was used for cDNA synthesis using First-strand cDNA Synthesis Kit (Thermo Fisher). Quantitative real-time RT-PCR using specific primer pair (Supplementary Table 4) for *Pm*STAT and WSSV immediate-early gene 1 (IE1) was employed to confirm the *Pm*STAT silencing efficiency, WSSV-challenged and immune-related gene response during WSSV infection by qRT-PCR. Real-time RT-PCR was carried out using an equal amount of cDNAs (2 µl of tenfold diluted cDNA) in the iCycler iQTM Real-Time detection system and the Luna® Universal qPCR Master Mix (Bio-Rad, USA). The qRT-PCR conditions were 95 °C for 30 s, followed by 40 cycles of 95 °C for 5 s and 55 °C for 10 s. The qRT-PCR was done in triplicate. The fold difference of mRNA transcription was calculated by 2^−∆∆CT^ method.

Statistical analysis was carried out using the IBM SPSS Statistics 22 program with one-way ANOVA followed by a post hoc test (Tukey’s). The result differences were considered significant at *P* < 0.05.

## Sample preparation for microbiome study

Total DNA from the intestines was isolated for microbiota study. To this end, the intestines of nine shrimps have been collected from each treatment group, pooling three intestines as a single sample. The DNA of whole intestines was extracted using the DNA by Quick-DNA Fecal/Soil Microbe Miniprep Kit (Zymo Research, USA). The Qubit™ dsDNA HS (Invitrogen, USA) assay was performed to quantify the DNA concentration. The DNA integrity was confirmed using 1% agarose gel electrophoresis. The V3-V4 regions were amplified from genomic DNA using the following conditions: 95 °C for 3 min (denaturation), followed by 25 cycles of 95 °C for 30 s, 55 °C for 30 s, and 72 °C for 30 s, and a final extension at 72 °C for 5 min. The PCR product was analyzed using 2% agarose gel by electrophoresis and purified using Ampure XP beads (Bechman Coulter). All purified amplicon samples were mixed in equal concentrations and sequenced on the Illumina Miseq sequencing platform using 250 base pair (bp) paired-end (PE) chemistry.

## Microbiome analysis

The results from sequencing were filtered and analyzed by Quantitative Insights Into Microbial Ecology (QIIME (version 1.8), http://qiime.org/index.html) as previously reported (Caporaso et al., 2010). The filtered sequences were assigned to the same operational taxonomic units (OTUs) with 97% similarity to the Green Genes reference sequence collection. Therefore, the taxonomic information was assigned to the Green Genes reference database. The OTUs at family, genera, and species levels were subjected to a LEfSe analysis to obtain the significantly different taxonomies among treatments with a significant level (alpha) of 0.05 and the LDA threshold > 2. For all data, statistical significance was set at *p* < 0.05. Alpha diversity index, including Chao1, Observed species, Phylogenetic diversity (PD), and Shannon diversity index, were calculated via QIIME to estimate diversity within the samples. The significant difference in alpha diversity index between groups was carried out using the IBM SPSS Statistics 22 program with one-way ANOVA followed by a post hoc test (Tukey’s). The result differences were considered significant at *P* < 0.01. Unweight UniFrac distances were calculated using the QIIME program and visualized using PCoA analysis to estimate the beta diversity. In addition, UPGMA (Unweighted Pair Group Method with Arithmetic mean) was analyzed by QIIME using the beta diversity distance matrix to compare between conditions. Finally, Anosim was performed to measure the differences in the bacterial profiles between groups. The resulting *P*-value indicated a significant difference between the two groups (*P*-value < 0.01). The distance between centroids of the PCoA (Fig. 3A) was determined with R using the "usedist" package and the matrix obtained of the beta diversity analysis with the unweighted UniFrac distances.

To find the presence of probiotics in our microbiome samples, we followed the analysis used by Ochoa-Romo et al.^45^. First, we obtained a new BIOM file from the joined sequences and clustered it with a 97% identity level against the Silva132 database. This database was used because it contains the reference of most probiotic bacteria. Afterward, the relative abundance of 70 probiotics taxa was extracted from the BIOM file using the summarize_taxa_throught_plots.py and an in-house script. Lastly, the Wilcoxon test was performed to determine the significant differential abundance of probiotics among tested groups.

## Supplementary Information

Supplementary Information.

## Data Availability

All data generated or analysed during this study are included in this published article and its supplementary information file.

## Abbreviations

*Pm*: *Penaeus monodon*
WSSV: White spot syndrome virus
