# Supplementary material for: White spot syndrome virus impact on the expression of immune genes and gut microbiome of black tiger shrimp Penaeus monodon
Source: Sci Rep. 2023 Jan 18;13:996. doi: 10.1038/s41598-023-27906-8 (PMC9849358; doi:10.1038/s41598-023-27906-8)
Supplement: Supplementary file 1 — Supplementary Information. [file 41598_2023_27906_MOESM1_ESM.docx]

**Supplementary Information**

**White spot syndrome virus impact on the expression of immune genes and gut microbiome of black tiger shrimp *Penaeus monodon***

Thapanan Jatuyosporn^1,2^, Pasunee Laohawutthichai^1,2^, Juan Pablo Ochoa Romo^3^, Luigui Gallardo-Becerra^3^, Filiberto Sánchez Lopez^3^, Anchalee Tassanakajon^2^, Adrián Ochoa-Leyva^3, *^ and Kuakarun Krusong^1, *^

^1^ Center of Excellence in Structural and Computational Biology, Department of Biochemistry, Faculty of Science, Chulalongkorn University, Bangkok, 10330, Thailand

^2^ Center of Excellence for Molecular Biology and Genomics of Shrimp, Department of Biochemistry, Faculty of Science, Chulalongkorn University, Bangkok, 10330, Thailand

^3^ Departamento de Microbiología Molecular, Instituto de Biotecnología (IBT), Universidad Nacional Autónoma de México (UNAM), Av. Universidad #2001, Col. Chamilpa, Cuernavaca, Morelos, 62210, Mexico.

* To whom correspondence should be addressed:

Kuakarun Krusong: Email: [Kuakarun.k@chula.ac.th](mailto:Kuakarun.k@chula.ac.th)

Adrián Ochoa-Leyva: Email: [adrian.ochoa@ibt.unam.mx](mailto:adrian.ochoa@ibt.unam.mx)

**
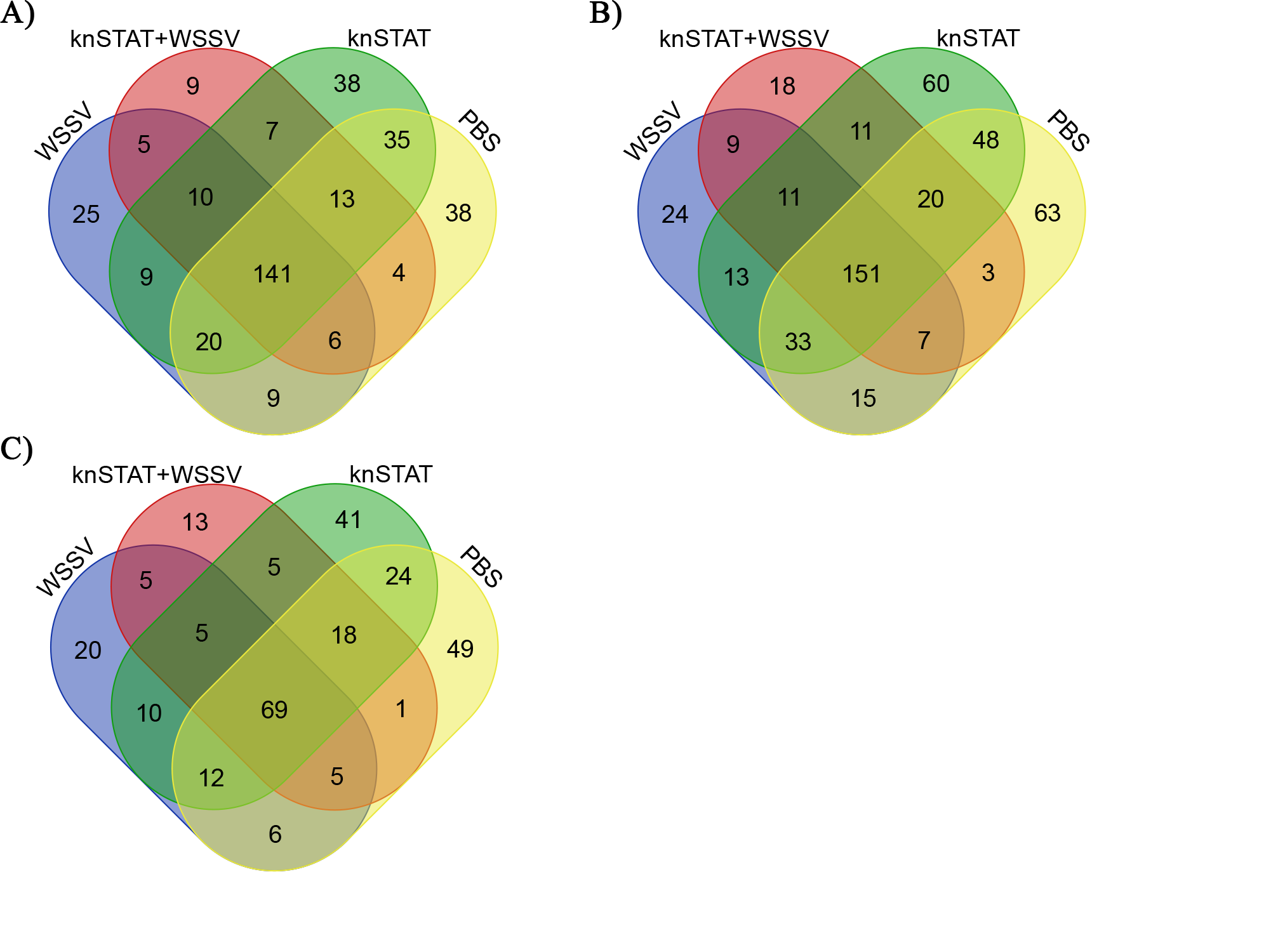
**

**Fig. S1** Venn diagram with shared (A) family, (B) genus and (C) species between intestine samples


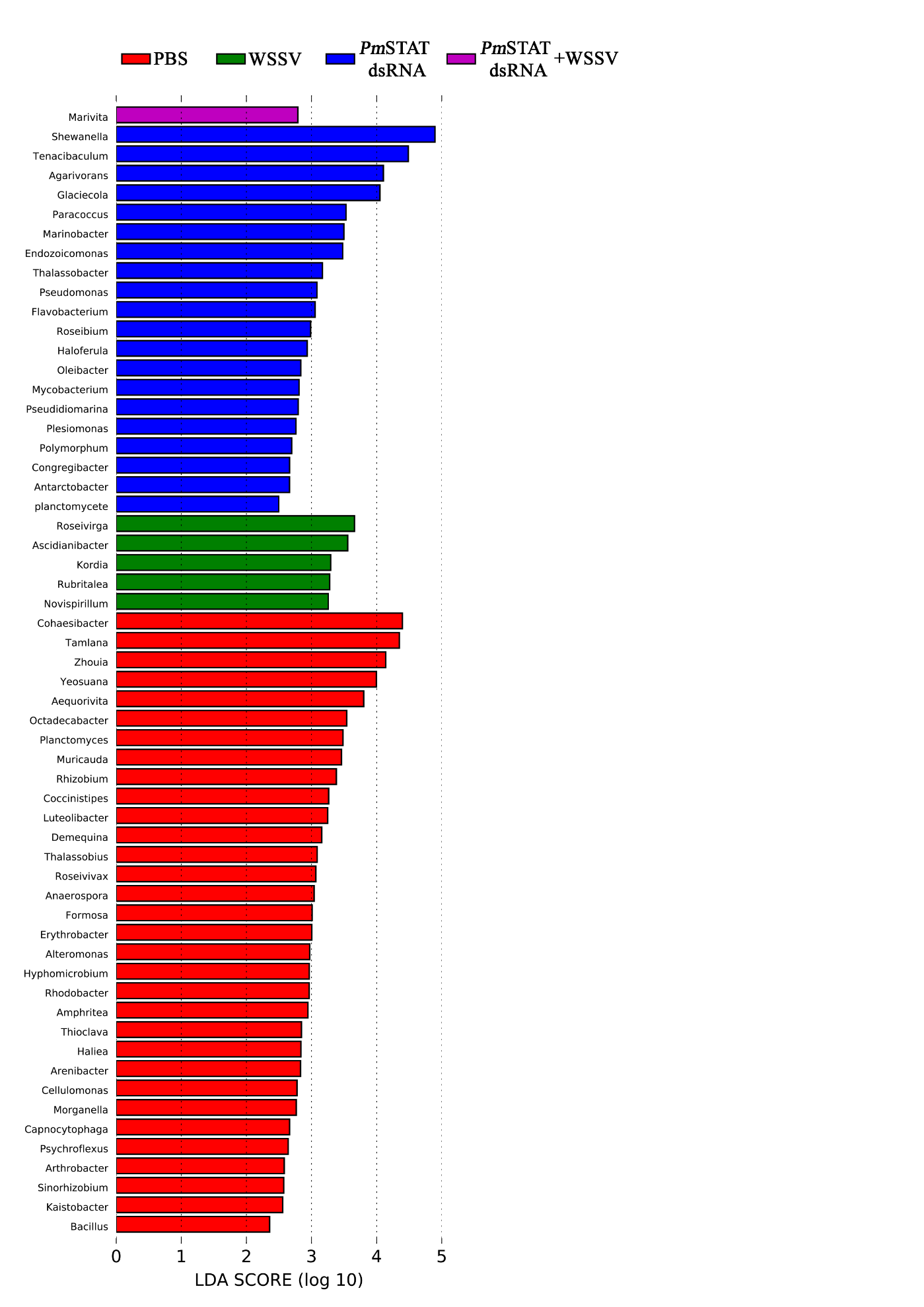


**Fig. S2** LEfSe analysis with the differentially abundant genera between groups of intestine samples


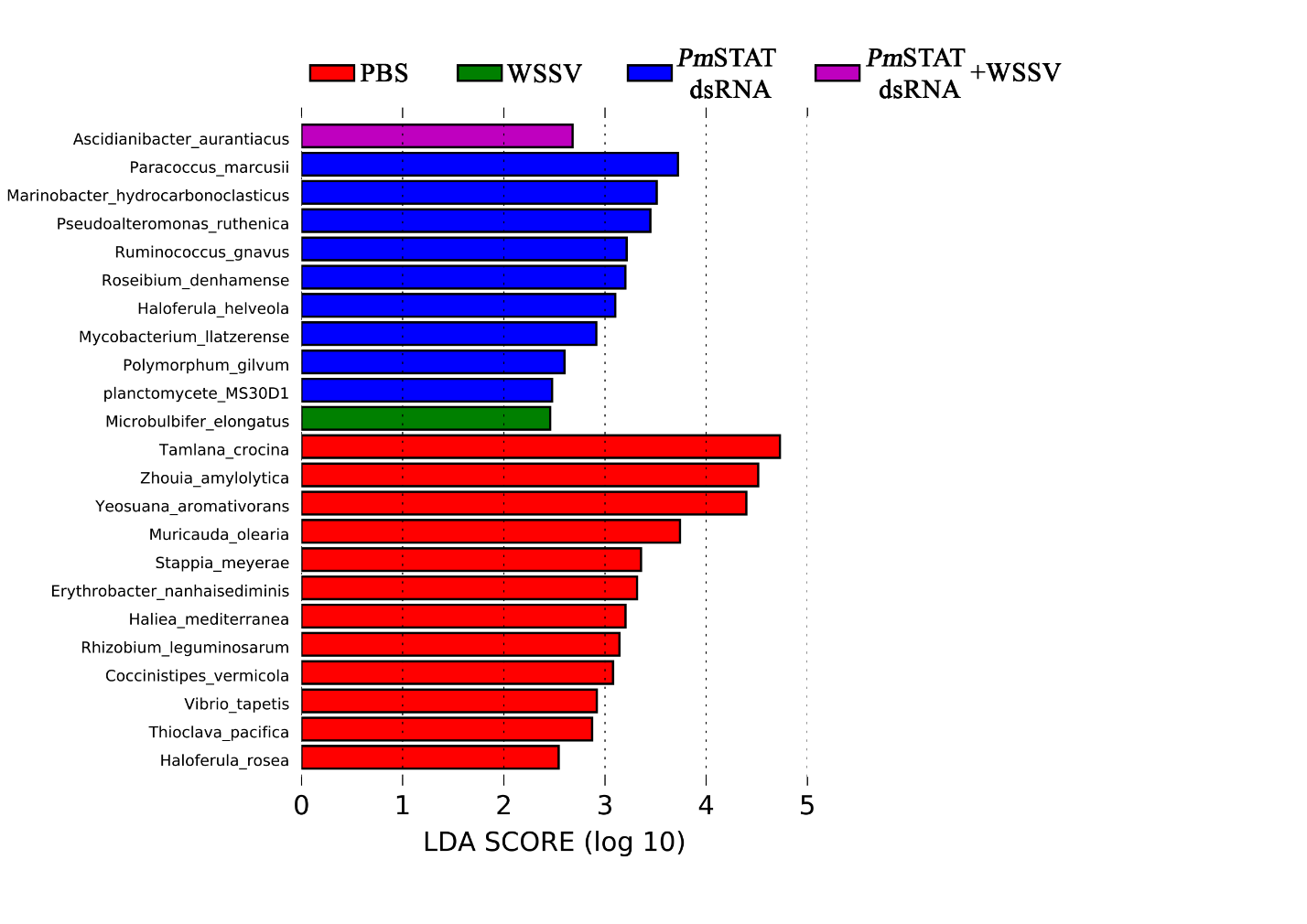


**Fig. S3** LEfSe analysis with the differentially abundant species between groups of intestine samples

**
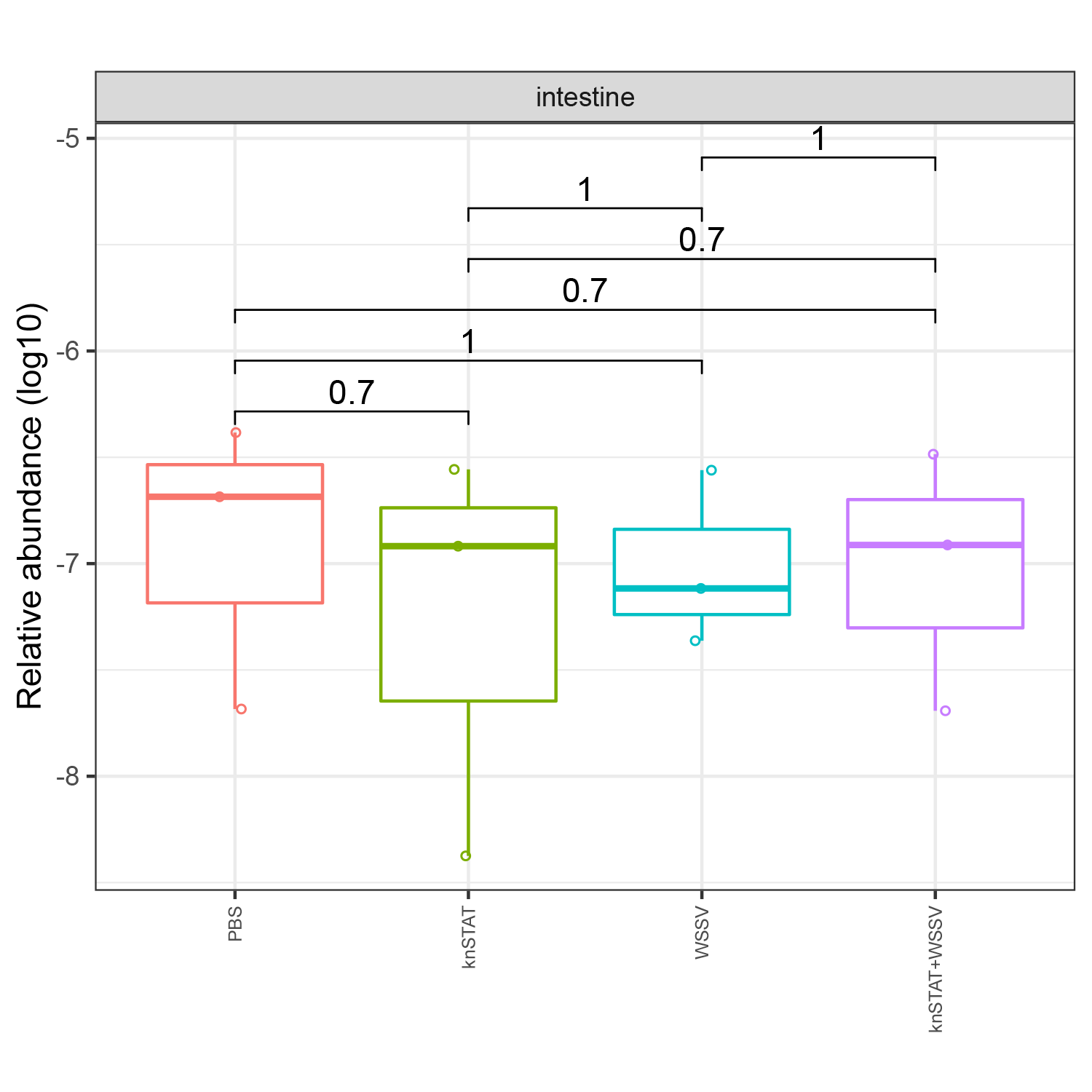
**

**Fig. S4** Overall intestinal probiotic relative abundance (log10) in PBS, *Pm*STAT silenced, WSSV challenged, and *Pm*STAT silenced + WSSV challenged shrimp.


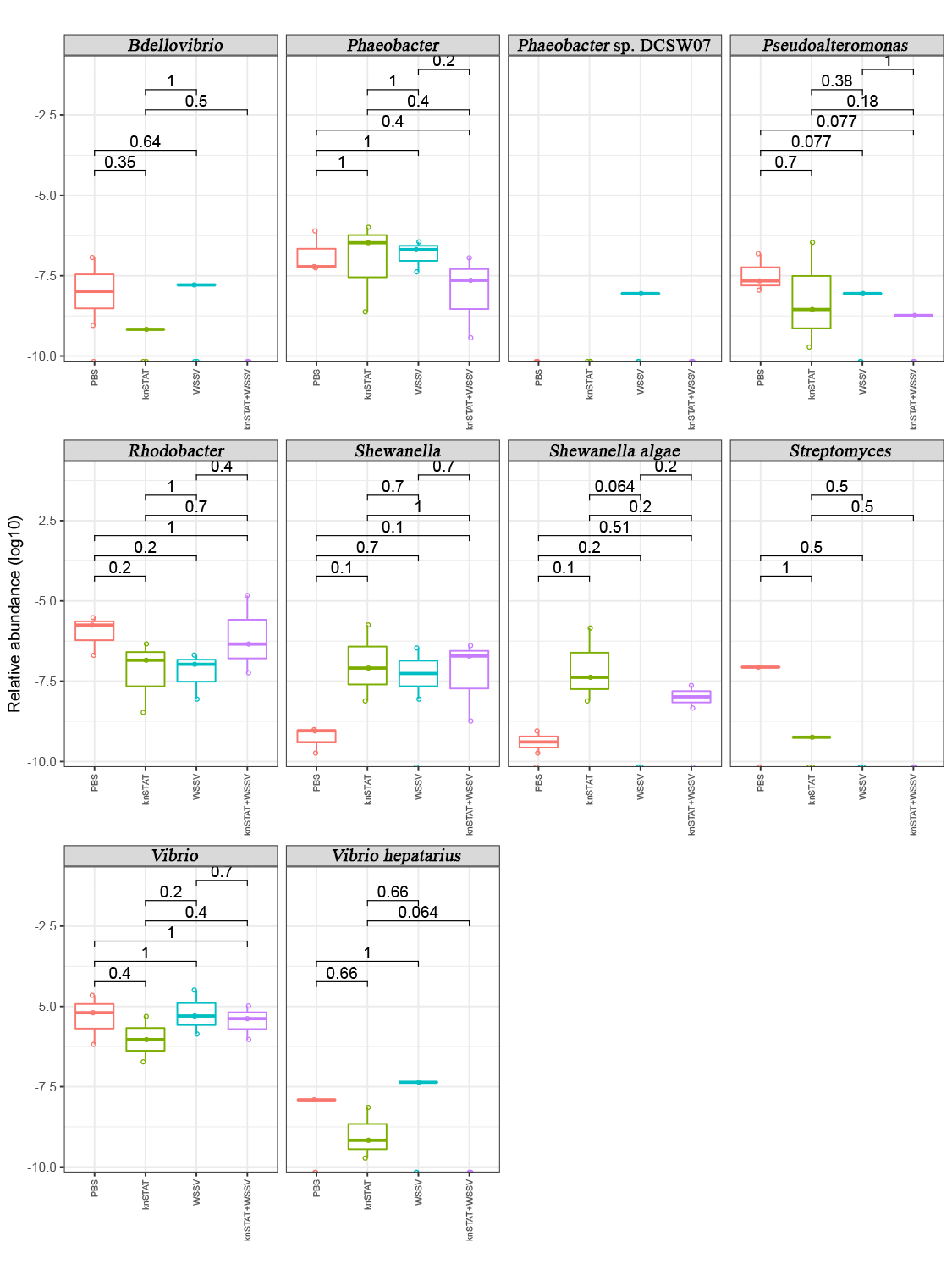


**Fig. S5** Intestinal probiotic relative abundance (log10) in PBS, *Pm*STAT silenced, WSSV challenged, and *Pm*STAT silenced + WSSV challenged shrimp.


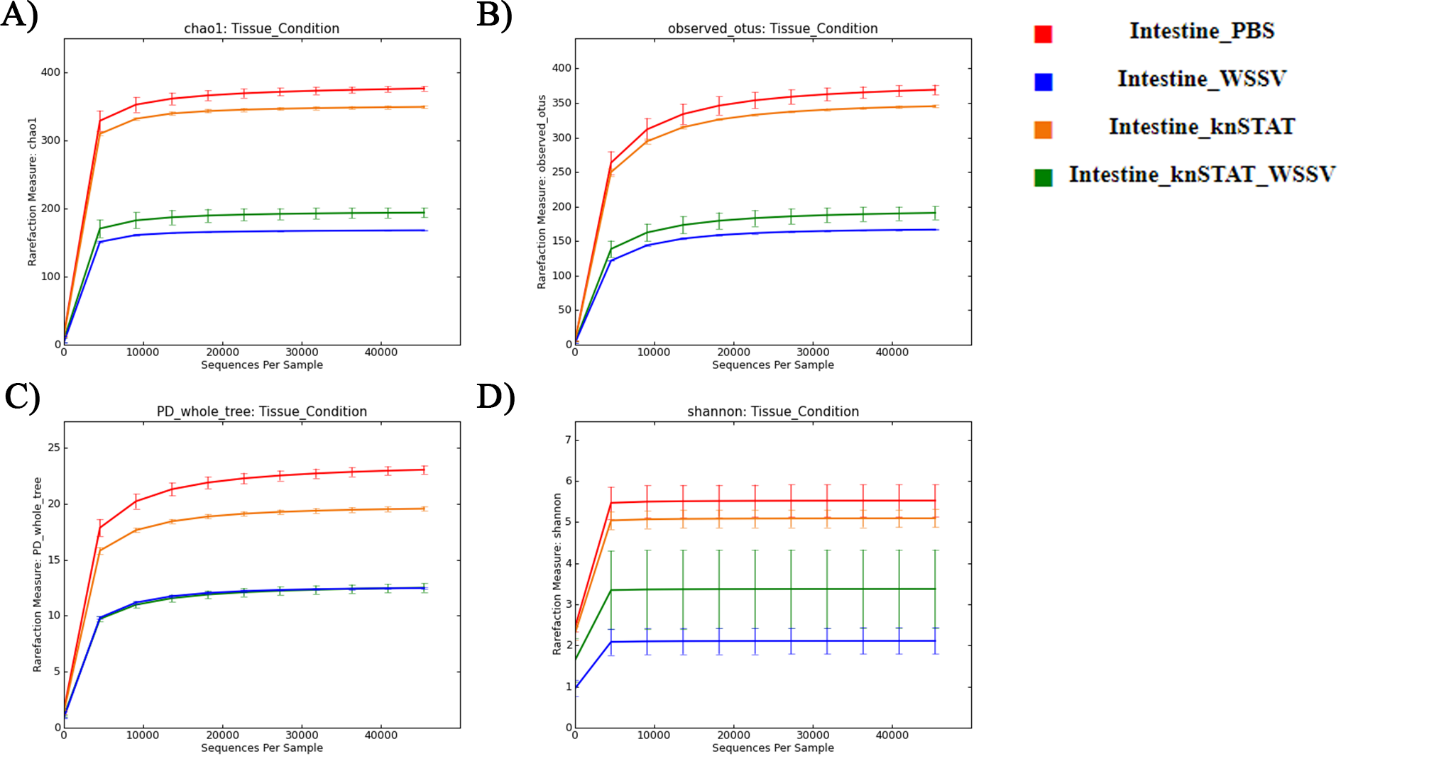


**Fig. S6** Alpha diversity rarefaction curves including (A) Chao1, (B) Observed OTUs, (C) PB and (D) Shannon diversity index in each condition.

**Table S1** Summary of total read processing

| Sample ID | Condition | Total read | Joined read | Split read | OTUs assigned read | Read without singletones |
| --- | --- | --- | --- | --- | --- | --- |
| K1 | PBS | 127,098 | 96,336 | 93,907 | 65,556 | 63,124 |
| K2 | PBS | 150,984 | 110,694 | 90,479 | 79,871 | 77,047 |
| K3 | PBS | 129,779 | 81,725 | 71,535 | 62,725 | 60,557 |
| K4 | *Pm*STAT dsRNA | 137,235 | 123,584 | 97,622 | 84,471 | 81,162 |
| K5 | *Pm*STAT dsRNA | 162,622 | 146,131 | 128,537 | 98,246 | 94,696 |
| K6 | *Pm*STAT dsRNA | 129,914 | 110,693 | 80,342 | 74,739 | 70,205 |
| K7 | WSSV | 117,755 | 103,987 | 91,557 | 88,929 | 86,966 |
| K8 | WSSV | 128,080 | 106,953 | 105,007 | 90,214 | 88,100 |
| K9 | WSSV | 101,376 | 107,417 | 84,922 | 77,904 | 75,820 |
| K10 | *Pm*STAT dsRNA +WSSV | 115,843 | 88,872 | 87,197 | 72,229 | 70,669 |
| K11 | *Pm*STAT dsRNA +WSSV | 120,471 | 91,313 | 78,124 | 74,050 | 72,490 |
| K12 | *Pm*STAT dsRNA +WSSV | 123,097 | 105,683 | 88,276 | 83,798 | 81,673 |

**Table S2** Summary of total read processing in percentage compared to total read

| Sample ID | Condition | Total read | Joined read | Split read | OTUs assigned read | Read without singletones |
| --- | --- | --- | --- | --- | --- | --- |
| K1 | PBS | 100.00% | 75.80% | 73.89% | 51.58% | 49.67% |
| K2 | PBS | 100.00% | 73.32% | 59.93% | 52.90% | 51.03% |
| K3 | PBS | 100.00% | 62.97% | 55.12% | 48.33% | 46.66% |
| K4 | *Pm*STAT dsRNA | 100.00% | 90.05% | 71.13% | 61.55% | 59.14% |
| K5 | *Pm*STAT dsRNA | 100.00% | 89.86% | 79.04% | 60.41% | 58.23% |
| K6 | *Pm*STAT dsRNA | 100.00% | 85.20% | 61.84% | 57.53% | 54.04% |
| K7 | WSSV | 100.00% | 89.77% | 79.04% | 76.77% | 75.07% |
| K8 | WSSV | 100.00% | 88.78% | 87.16% | 74.88% | 73.13% |
| K9 | WSSV | 100.00% | 87.26% | 68.99% | 63.29% | 61.59% |
| K10 | *Pm*STAT dsRNA +WSSV | 100.00% | 75.47% | 74.05% | 61.34% | 60.01% |
| K11 | *Pm*STAT dsRNA +WSSV | 100.00% | 71.29% | 61.00% | 57.82% | 56.60% |
| K12 | *Pm*STAT dsRNA +WSSV | 100.00% | 85.21% | 71.18% | 67.57% | 65.85% |

**Table S3** Percentage of assigned reads without unclassified taxonomic

| Sample ID | Condition | Phylum | Class | Order | Family | Genus | Species |
| --- | --- | --- | --- | --- | --- | --- | --- |
| K1 | PBS | 100.00% | 100.00% | 99.60% | 95.91% | 49.54% | 16.63% |
| K2 | PBS | 100.00% | 100.00% | 98.79% | 95.88% | 67.09% | 40.05% |
| K3 | PBS | 100.00% | 100.00% | 99.07% | 92.02% | 45.26% | 22.64% |
| K4 | *Pm*STAT dsRNA | 100.00% | 100.00% | 98.78% | 97.96% | 75.82% | 54.34% |
| K5 | *Pm*STAT dsRNA | 100.00% | 100.00% | 97.61% | 96.41% | 66.16% | 33.89% |
| K6 | *Pm*STAT dsRNA | 100.00% | 100.00% | 69.85% | 68.41% | 47.92% | 19.55% |
| K7 | WSSV | 100.00% | 100.00% | 99.75% | 98.61% | 91.84% | 84.46% |
| K8 | WSSV | 100.00% | 100.00% | 99.85% | 99.31% | 88.68% | 75.52% |
| K9 | WSSV | 100.00% | 100.00% | 99.88% | 98.63% | 88.19% | 73.57% |
| K10 | *Pm*STAT dsRNA +WSSV | 100.00% | 100.00% | 98.80% | 97.90% | 81.53% | 51.31% |
| K11 | *Pm*STAT dsRNA +WSSV | 100.00% | 100.00% | 99.67% | 98.42% | 88.44% | 78.84% |
| K12 | *Pm*STAT dsRNA +WSSV | 100.00% | 100.00% | 99.63% | 98.54% | 71.55% | 12.35% |

**Table S4** Nucleotide sequences of the primers

| Primer name | Sequence (5’ – 3’) | Experiment | References |
| --- | --- | --- | --- |
| dsSTAT-T7-F | GGATCCTAATACGACTCACTATAGG  GCCAGTTGTAGTCATTGTCC | double-stranded RNA synthesis | Wen *et al.*, 2014 |
| dsSTAT-F | GCCAGTTGTAGTCATTGTCC | double-stranded RNA synthesis |  |
| dsSTAT-T7-R | GGATCCTAATACGACTCACTATAGG  CAAAGCTGCCACTGGAAGGG | double-stranded RNA synthesis |  |
| dsSTAT-R | CAAAGCTGCCACTGGAAGGG | double-stranded RNA synthesis |  |
| *Pm*STAT-qRT-F | TATATCCGAATGTGCCTAAG | real-time RT-PCR |  |
| *Pm*STAT-qRT-R | ATAGTTTGTGGTGTGTTGGG | real-time RT-PCR |  |
| EF1-α-F | GGTGCTGGACAAGCTGAAGGC | real-time RT-PCR | Jatuyosporn *et al.*, 2019 |
| EF1-α-R | CGTTCCGGTGATCATGTTCTTGATG | real-time RT-PCR |  |
| *Pm*Spätzle-qRT-F | TAAGCAAGGAGCAGGAAGAG | real-time RT-PCR | Boonrawd *et al.*, 2017 |
| *Pm*Spätzle-qRT-R | TGGCATACACCACATCTGAG | real-time RT-PCR |  |
| *Pm*Dorsal-qRT-F | TCACTGTTGACCCACCTTAC | real-time RT-PCR | Visetnan *et al.*, 2015 |
| *Pm*Dorsal-qRT-R | GGAAAGGGTCCACTCTAATC | real-time RT-PCR |  |
| *Pm*Relish-qRT-F | TCTCCAGGTGAGCACTCAGTTGGC | real-time RT-PCR |  |
| *Pm*Relish-qRT-R | GCTGTAGCTGTTGCTGTTGTTGAG | real-time RT-PCR |  |
| *Pm*DOME-F | CTCAGGCTATGTTTCTCAGGATTCA | real-time RT-PCR | Jatuyosporn *et al.*, 2019 |
| *Pm*DOME-R | CACGGCAGTTCCTTTATGGTCT | real-time RT-PCR |  |
| *Pm*PEN3-F | GGCTTAGCCCCTTACA | real-time RT-PCR | Soponpong *et al.*, 2018 |
| *Pm*PEN3-R | GACCCATACCTACAAATAAC | real-time RT-PCR |  |
| Crustin*Pm*1-F | CTGCTGCGAGTCAAGGTATG | real-time RT-PCR | Arayamethakorn *et al.*, 2017 |
| Crustin*Pm*1-R | AGGTACTGGCTGCTCTACTG | real-time RT-PCR |  |
| Crustin*Pm*7-F | GGCATGGTGGCGTTGTTCCT | real-time RT-PCR |  |
| Crustin*Pm*7-R | TGTCGGAGCCGAAGCAGTCA | real-time RT-PCR |  |
| *Pm*JAK-F | TGCTGTTCCGACTGCGTTTC | real-time RT-PCR | Jatuyosporn *et al.*, 2019 |
| *Pm*JAK-R | GCGTGGAAGTCTGCTCGAAC | real-time RT-PCR |  |
| *Pm*MyD88-F | GTGCACCAGAGTCATTGTAG | real-time RT-PCR | Arayamethakorn *et al.*, 2017 |
| *Pm*MyD88-R | GGGAGTGGCAGAAACTTATC | real-time RT-PCR |  |
| WSSV-IE1-F | GCTAGGGATGTGACTTTC | real-time RT-PCR | Jatuyosporn *et al.*, 2019 |
| WSSV-IE1-R | TGCACCTACACGCATTAC | real-time RT-PCR |  |
| *Pm*ProPO1-F | GGTCTTCCCCTCCCGCTTCG | real-time RT-PCR | Amparyap *et al.*, 2012 |
| *Pm*ProPO1-R | GCCGCAGGTCCTTTGGCAGC | real-time RT-PCR |  |
| *Pm*ProPO2-F | GCCAAGGGGAACGGGTGATG | real-time RT-PCR |  |
| *Pm*ProPO2-R | TCCCTCATGGCGGTCGAGGT | real-time RT-PCR |  |
| *Pm*PPAE1-F | ATGAAGGGCGTGACGGTGGTTCTATG | real-time RT-PCR |  |
| *Pm*PPAE1-R | CTCTTCTTCAAGCTCACCACTTCTATCT | real-time RT-PCR |  |
| *Pm*PPAE2-F | ATGCACTACCGGGTTCCCACGATC | real-time RT-PCR |  |
| *Pm*PPAE2-R | CTAAGGTTTGAGATTCTGCACG | real-time RT-PCR |  |
| *Pm*Vago1-F | GAACACACCCCAGTGCACTGGT | real-time RT-PCR | Nhnhkorn *et al.*, 2019 |
| *Pm*Vago1-R | ATGGAGCTTGTTCCCCTTCTGTG | real-time RT-PCR |  |
| *Pm*Vago4-F | ACTCCTCTCCCTTCAGGGCATC | real-time RT-PCR |  |
| *Pm*Vago4-R | TGGCAGGAACTTCTCTCGCTGC | real-time RT-PCR |  |
| *Pm*Vago5-F | AGAAGCATTTAGGCTCAGGGCAG | real-time RT-PCR |  |
| *Pm*Vago5-R | GATGGCCAGAGTTATTGTGACGC | real-time RT-PCR |  |
| *Pm*IKKβ-F | CTGAGGGCATGACGCGACCAC | real-time RT-PCR |  |
| *Pm*IKKβ-R | GCCTGCTCATCATAGTAGTCGAG | real-time RT-PCR |  |
| *Pm*IKKε1-F | GTCACCCAGTTAACGACTCTGCA | real-time RT-PCR |  |
| *Pm*IKKε1-R | TGCTGATTGGGTGGGTGATGAC | real-time RT-PCR |  |
| *Pm*IKKε2-F | GTCACCCAGTTAACGACTCTGCA | real-time RT-PCR |  |
| *Pm*IKKε2-R | GCATTGAGGGAATCACGAACAGCT | real-time RT-PCR |  |
| *Pm*Castus-F | ACGGCAGTAGGATCGGGGTTTGCCT | real-time RT-PCR | Arayamethakorn *et al.*, 2017 |
| *Pm*Castus-R | ATGCCCCACAGAGGTGATGCCCTGA | real-time RT-PCR |  |

The underline sequence present T7 promotor sequence use in dsRNA generating experiment.
